# Applying GC-MS analysis to identify chemical composition of Iranian propolis prepared with different solvent and evaluation of its biological activity

**DOI:** 10.22088/cjim.11.2.191

**Published:** 2020

**Authors:** Fariba Asgharpour, Ali Akbar Moghadamnia, Sohrab Kazemi, Hamid Reza Nouri, Mina Motallebnejad

**Affiliations:** 1 Dental Materials Research Center, Health Research Institute, Babol University of Medical Sciences, Babol, Iran; 2 Neuroscience Research Center, Health Research Institute, Babol University of Medical Sciences, Babol, Iran; 3 Cellular and Molecular Biology Research Center, Health Research Institute, Babol University of Medical Sciences, Babol, Iran; 4 Oral Health Research Center, Health Research Institute, Babol University of Medical Sciences, Babol, Iran

**Keywords:** Cytotoxicity, Flavonoids, Gas chromatography-mass spectrometry, Propolis

## Abstract

**Background:**

Propolis as a natural product has shown beneficial effects on human health. This study was aimed to investigate the chemical compositions and biological activity of three different extracts of propolis from two distinct geographic areas in Iran.

**Methods:**

The chemical composition of Iranian propolis extracts that were collected in the Spring of 2016 from two provinces in northern Iran: Ardabil and Polur in Mazandaran Province were measured through gas chromatography-mass spectrometry (GC-MS) methods. In addition, antimicrobial activity and cytotoxicity effect on HN5 and LNCaP cell lines were evaluated. The data were analyzed using one-way ANOVA and p<0.05 was considered as significant.

**Results:**

The GC-MS analysis identified the presence of compounds that belonged to the different groups such as aromatics acids and their related esters, flavonoid and flavonoid derivatives and terpenes. Flavanone was the most dominant compound of flavonoids. The maximum growth inhibition was observed against *S. aureus* of ethanolic extract of propolis (p<0.05). Moreover, cytotoxicity showed that ethanolic and dichloromethane extracts had more inhibitory effects on cell lines than the water extract.

**Conclusion:**

The results determined that extracts had the highest percentage of flavonoids. Therefore, it is expected that the synergistic effect of the main components of propolis is related to the increase of biological activity of propolis.

Propolis is a wax-like substance produced by honey bees. It is soft and can be found in colors ranging from yellowish-green to dark brown (1-3). Propolis acts as a disinfectant for bees and an effective agent for preventing the incidence and outbreak of diseases in the beehive (4, 5). Propolis is generally composed of about 50% resins, 30% beeswax, 10% essential fatty acids, 5% pollen, and 5% other organic compounds including vitamins, and minerals. The amount and type of constituting compounds depends on the location and time of collection (1, 6). Different types of resins such as poplars, conifers, birch, pine, alder, willow, and palm were identified in propolis. The best-known variety of propolis is poplar propolis. This propolis is mainly composed of flavonoids, phenolic acids, and their esters, which distinguish it from other propolis. The main compounds associated with the biological activity of propolis include polyphenols, aromatic acids, and diterpenic acids (7-9). The solvents commonly used in propolis extraction are water, methanol, ethanol, chloroform, dichloromethane, ether, and acetone (1, 10). Ethanol is the most used solvent to obtain low-wax propolis extracts that are rich in active biological compounds (11). 

With the advancements in separation and purification techniques, other compounds in propolis, including flavonoids, terpenes, phenols and esters, sugars, hydrocarbons, and mineral elements have been identified (10). Flavonoids exhibit a wide range of biological properties and depending on their chemical composition can be classified into flavones, flavonols, flavanones, chalcones, dihydrochalcones, isoflavones, isodihydroflons, flavanes, isoflavanes, and neoflavonoids (12,7). Propolis is known to have positive effects on human health and has been used in traditional medicine since ancient times (13). Studies carried out on propolis extracts from different parts of the worlds have demonstrated the antimicrobial, anti-inflammatory, cytotoxic, and antiparasitic properties and immunomodulatory and anti-leishmanicidal effects (14, 15). Given the potential applications of propolis, this study used the GC/MS analysis method to investigate the chemical composition of ethanol, dichloromethane, and water extracts of propolis of northern Iran and compared the effect of different solvents on biological activity of the extract.

## Methods

**Propolis samples: **This study was approved by the Research Ethics Committee of Babol University of Medical Sciences (MUBABOL.REC.1394020). Samples of crude propolis produced by *Apis mellifera* bees were collected in the Spring of 2016 from Sabalan Mountains in Ardabil Province (Ardabil city) and Alborz Mountains in Mazandaran Province (Polur). Ethanolic extract of Iranian propolis (EEIP), dichloromethane extract of Iranian propolis (DEIP) and water extract of Iranian propolis (WEIP) were prepared according to the 2018 study by Afrouzan et al. (16).

**GC/MS analysis: **The EEIP, DEIP and WEIP of propolis samples were analyzed using gas chromatography–mass spectrometry (GC–MS) equipment (7890B-5977B MSD, Agilent). The experimental conditions for the DB-5 MS capillary column were as follows: length=30 m; ID=0.25 mm; film thickness = 0.25 μm; and the carrier gas was helium at a flow rate of 1 ml/min. A sample of 1 μl was injected with an auto sampler in a split ratio of 10:1. The injector temperature was set at 250 °C and the oven temperature was programmed from 50 °C (storage time of 1 min) and increased at a rate of 8°C/min to 120 °C (storage time of 1 min), and then increased at a rate of 6 °C/min to 250°C, finally to 250°C at 15 min. The solvent delay was 0 to 3 min, and the total GC–MS running time was 47 min. Using the National Institute of Standards and Technology (NIST 11 Variant) database, the mass spectrum was used to identify the name, molecular weight, and structure of the components of propolis samples.

**Antibacterial activity: **Gram-positive strain includes *staphylococcus aureus *(ATCC 25923), and gram-negative strains include *Escherichia coli *(ATCC 25922) and *Pseudomonas aeruginosa* (ATCC 15442), which were provided in lyophilized form by Iranian Research Organization for Science and Technology (IROST). Lyophilized culture of strains was transferred from the stock culture to Brain Heart Infusion (BHI) and Muller Hinton agar (MHA) (Merck, Darmstadt, Germany) and incubated at 37 °C for 24 h. Suspensions of bacteria was prepared according to the turbidity of 0.5 McFarland turbidity (17). The broth micro-dilution method was used to determine minimum inhibitory concentration (MIC) of extracts according to the Clinical and Laboratory Standards Institute guidelines (18). 

**Cell cultures and cytotoxicity analysis: **The cytotoxic effect of extracts was tested on cancer cell lines including the human prostate cancer cell line (LNCaP) and the head and neck carcinoma cell line (HN5). LNCaP and HN5 cell line was obtained from the National Cell Bank of Iran, Pasteur Institute (Tehran, Iran) and was cultured in RPMI 1640 containing L-glutamin and supplemented with 10% fetal calf serum (FBS), and 1% Penstrep (penicillin G 100 IU/ml, streptomycin 100 μg/ml). The cell lines were grown as monolayers in 25 cm^2 ^cell culture flasks at 37°C in a 5% CO2 humidified atmosphere. Cells were treated with 0 up to 500 μg/ml of EEIP, DEIP and WEIP. Each concentration was tested in triplicates along with the control group (without treatment). The cytotoxic effect was measured using the MTT assay after 48 h incubation (12). The obtained OD from the control group was considered as 100% viability.

**Statistical analysis: **Data were analyzed using GraphPad Prism v 6.07 (GraphPad Software Inc., La Jolla, CA, USA). Results were expressed as the mean± SD. Comparisons between groups was performed via one-way ANOVA. P<0.05 was considered statistically significant.

## Results

In particular, EEIP extract of Ardabil showed the compounds of different aromatic acids and their related esters such as benzeneethanol, 3-hydroxy-4-methoxycinnamic acid, 5-phenylthiazolidine and flavonoid (galangin flavanone) and derivatives (pinostrobin chalcone, techtochrysin). Also, it indicated the terpene derivatives (dihydro-. alpha. -terpineol). EEIP extract of Polur had different aromatic acid and corresponding esters such as benzeneethanol, dihydrobenzofuran, 4-hydroxycinnamic acid, cinnamic acid, 4-hydroxy-3-methoxy-, 2,5 dimethoxyterephthalic acid, 3- hydroxy-4-methoxycinnamic acid, methyl 3-(4'-hydroxyphenyl)prop-2-enoate, 1,4- Dihydrophenanthrene, diethylmethylbenzyloxysilane, benzeneacetic acid, methyl ester, as well as flavonoid and flavonoid derivatives (chrysin, pinostrobin chalcone, 5,7 -dihydroxy -dihydroflavone, tectochrysin). The combination of terpenes was not found (table 1).

**Table 1:** Compounds identified the extracts of Iranian propolis using GC/MS analysis

^a^ ** R.T. min**	**Compounds**	**Composition (%)** * EP EA DP DA WP WA					
5.642	Dimethyl sulfone	-	-	-	-	-	5.882
9.144	Benzeneethanol	3.312	3.004	-	-	-	-
11.177	Dihydrobenzofuran	1.662	-	-		8.45	3.411
13.327	2-Methoxy-4-vinylphenol	-	-	-	-	1.774	-
16.057	2,2-Diethynylbut-2-ene-1,4-diol	15.663	-	-	-	85.552	82.894
20.313	-4-Methyl-2-(1-ethylethenyl)-1-cyclopentene-1-carboxaldehyde	-	1.379	-	-	-	-
20.365	3-Ethyl-8-methyl-2-oxatetracyclo[4.4.0.0(1,4).0(6,8)]decane	-	1.370	-	-	-	-
23.260	Dihydro-.alpha.-terpineol	-	3.273	-	-	-	-
23.276	Rosifoliol	1.549	-	-	-	-	-
26.035	(-)-Elema-1,3,11(13)-trien-12-al	-	2.104	-	-	-	-
26.053	1,3,6-Octatriene, 3,7-dimethyl-, (E)-	1.061	-	-	-	-	-
27.065	3,4-Octadiene, 7-methyl-	-	3.805	-	-	-	-
27.667	9-Dodecenol	-	-	-	0.765	-	-
27.672	Heptylacetylene	-	-	2.085	-	-	-
27.746	9-Octadecenoic acid (Z)-, methyl ester	-	4.805	-	-	-	-
28.742	Trifluoroacetic acid, n-heptadecyl ester	-	3.780	-	-	-	-
28.753	4-Hydroxycinnamic acid	2.549	1.395	-	-	-	-
29.448	Cinnamic acid, 4-hydroxy-3-methoxy-	1.003	-	-	-	1.233	4.264
29.961	2,5 Dimethoxyterephthalic acid	1.023	-	-	-	-	-
29.962	Benzaldehyde, 4,6-dimethoxy-2,3-dimethyl-	-	-	-	0.809	-	-
30.219	3- Hydroxy-4-methoxycinnamic acid	0.931	4.925	-	0.954	-	-
30.592	Methyl 3-(4'-hydroxyphenyl)prop-2-enoate	0.834	-	-	0.609	-	-
31.733	Aniline, 2,4,6-trimethyl-3-nitro-	-	-	-	12.975	-	-
31.785	1,4- Dihydrophenanthrene	7.883	-			-	-
32.172	Pinostrobin chalcone	12.820	15.051	19.710	8.595	-	-
32.253	3-Methoxy-4,5-methylenedioxybenzaldehyde	-	-	-	1.586	-	-
32.276	Diethylmethylbenzyloxysilane	0.877	-	-	-	-	-
32.408	Caffeic acid	-	-	-	5.619	-	-
33.148	p-Pentyloxynitrobenzene	-	-	1.352	-	-	-
33.426	5,7- -dihydroxy -dihydroflavone	16.855	-	-	-	-	-
33.359	Galangin flavanone	-	36.672	57.069	36.548	-	-
34.201	1,2-Benzenedicarboxylic acid	-	-	-	-	4.243	3.55
34.771	Hydrocinnamic acid	-	-	1.423	2.489	-	-
34.784	Methyl phenylacetate	-	-	1.094	-	-	-
34.817	Benzeneacetic acid, methyl ester	2.899	-	-	-	-	-
35.059	10-hydroxybenzo[j]fluoranthene	-	-	16.458	-	-	-
35.145	Tectochrysin	13.571	14.792	-	9.662	-	-
36.692	Naringenin	-	-	-	9.892	-	-
36.726	Chrysin	13.547	-	-	9.493	-	-
39.940	5-phenylthiazolidine	-	3.534	-	-	-	-

^a^RT: Retention time (minutes). * EP: ethanolic extract of polur, EA: ethanolic extract of Ardabil, DP: dichloromethane extract of polur, DA: dichloromethane extract of Ardabil, WP: water extract of polur, WA: water extract of Ardabil

**Figure 1:**
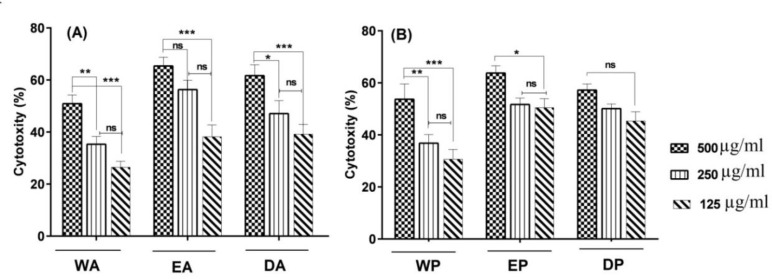
The cytotoxity of HN5 cell line was measured via MTT assays. The cells were treated with various concentrations for 48 h. The results are presented as a percentage of the control group. The data shown are the mean± SD of three determinations. * (p <0.05), * * (p <0.01), *** (p <0.001) and ns; non-significant

**Figure 2:**
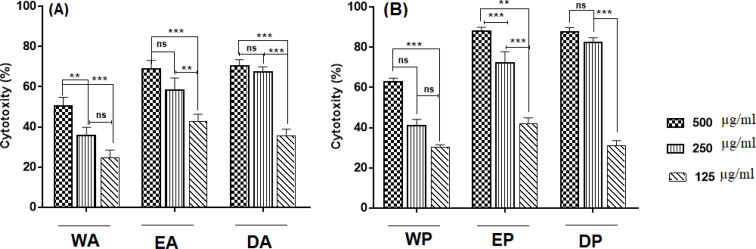
The cytotoxity of LNCaP cells was measured via MTT assays. The cells were treated with various concentrations for 48 h. The results are presented as a percentage of the control group. The data shown are the mean± SD of three determinations. * * (p <0.01), *** (p <0.001) and ns; non-significant

Regarding the DEIP extract of Ardabil propolis, the highest quantity compounds were flavonoids (pinostrobin chalcone, galangin flavanone, naringenin, tectochrysin, and chrysin). Other compounds were aromatic acids and its derivatives (benzaldehyde, 4,6-dimethoxy-2,3-dimethyl-, 3-hydroxy-4-methoxycinnamic acid, aniline, 2,4,6-trimethyl-3-nitro-, 3-methoxy-4,5-methylenedioxybenzaldehyde, caffeic acid and hydrocinnamic acid). Concerning Polur propolis, two types of flavonoids (galangin flavanone and pinostrobin chalcone) had the highest amounts, and the other identified compounds were aromatic acids (p-pentyloxynitrobenzene, hydrocinnamic acid, 10-hydroxybenzo[j]fluoranthene (table 1). In WEIP extract of Ardabil propolis, 2-ethenyl-1,3-benzenediol of aromatic acid had the highest quantity. In case of polur propolis, 85.552% of total was 2,2-diethynylbut-2-ene-1,4-diol. Flavonoids were not found in WEIP extract (table 1). According to these results, the all extracts were composed of aromatic acids and its related esters, flavonoid and flavonoid derivatives. EEIP of Ardabil propolis had little amounts of aromatic acids (11.463% of total) and more than flavonoid and flavonoid derivatives (66.515% of total) compared with Polur propolis 24.908% and 56.793% respectively. Tectochrysin and pinostrobin chalcone were common flavonoids identified in both extracts. In the DEIP extracts, Ardabil propolis had the highest content of flavonoids (76.779% of total). Also, galangin flavanone was the highest quantity compounds in samples. However, ethanolic extract was more efficient solvent for the isolation of phenolic compounds compared to other solvents. In MIC assay, DEIP and EEIP extract of Ardabil propolis at lower concentrations were able to inhibit *S. aureus *and* E. coli *compared with Polur propolis. The all extracts (up to a concentration of 1000 µg/ml) were not able to inhibit the growth of *P.aeruginosa* (table 2). Our data showed that EEIP, DEIP and WEIP were able to induce cytotoxicity in a dose-dependent manner. Significant cytotoxic effects of propolis on HN5 and LNCaP cell lines are shown in figures.1 and 2. 

In the presence of 500 µg/ml of EEIP propolis revealed the highest cytotoxicity on HN5 cell line, the percentage of cell viability decreased to 34.6% for EEIP of Ardabil (EA) and 36.2% for EEIP of Polur (EP) compared to control group (without treatment). Also, 500 µg/ml of EP revealed the highest cytotoxicity on LNCaP cells (87.8 %). A significant difference was found between EA and EP in concentrations of 500 µg/ml and 125 µg/ml (P<0.001) and between DA and DP in concentrations of 500 µg/ml and 250 µg/ml (P<0.01).

**Table 2:** The minimum inhibitory concentration (MIC) values of extracts against strains (values in µg/ml)

	**The Minimum Inhibitory Concentration (MIC) µg/ml**									
**Samples**	***S. aureus*** ** (ATCC 25923)**				***E. coli (*** **ATCC 25922** ***)***			***P.aeruginosa*** ** (ATCC 15442)**		
	**EEIP**	**DEIP**	**WEIP**		**EEIP**	**DEIP**	**WEIP**	**EEIP**	**DEIP**	**WEIP**
Ardabil	250 ^a^	250^ a^	1000^ b^		500	500	-	-	-	-
Polur	250^a^	500^ b^	1000^ b^		1000^a^	500^b^	-	-	-	-
Gentamicin	250	250	250		500	250	500	1000	500	1000
Chloramphenicol	1	4	8		4	8	8	64	64	128

Values are mean of three different tests. Means followed by the same letters are not significantly different in each row for each isolates (p<0.05), - >1000 µg/ml.

## Discussion

Considering the use of propolis in the industry, such as food, cosmetics and pharmaceutical industries, the analysis of physicochemical composition of propolis has been considered to determine the quality of this material. The chemical compositions of EEIP, DEIP and WEIP from two Iranian propolis samples were analyzed by GC-MS and showed the presence of compounds that belonged to different groups such as aromatic acids and their related esters, flavonoid and flavonoid derivatives and terpenes. 

The results showed that extraction solvent could play an important role in the isolation of bioactive compounds. In this concern, individual compounds were identified in ethanoic extract of Iranian propolis was more than the other solvents. However, both ethanolic and dichloromethane solvents showed a high percentage of flavonoids. Afrouzan et al. analyzed the chemical compositions from four Iranian propolis samples (Morad Beyg, Taleghan, Kalaleh and Chenaran) using GC-MS methods and indicated that the total amount of flavonoids in dichloromethane extracts of propolis was higher than ethanolic extracts (16). Alizadeh et al. investigated the chemical components of Iranian propolis from two origins of Hamadan and Taleghan and showed different amount of aromatic acids )2270 µg/ml and 489 µg/ml respectively) while the amount of phenolic compounds in the two areas was almost the same (1238 µg/ml and 1568 µg/ml, respectively). This difference between levels of aromatic compounds probably related to their geographical origins. Caffeic acid isoprenyl esther (isomer 2) and pinobanksin were the highest quantity compounds found in Hamadan and Taleghan propolis (32.52% and 16.52%, respectively) (19). The chemical components of ethanolic and water extracts of Iranian propolis from Kordkoy indicated that extracts have a high percentage of flavonoids, though the amount of flavonoids in ethanolic extract (15.88%) was more than water extracts (14.87%) (20). 

These studies indicated that Iranian propolis is rich in flavonoids and phenolic compounds. These diversity of flavonoid compounds showed the typical pattern of “poplar” propolis (21, 22). Analysis of Ardabil and polur propolis by HPLC in the previous study showed the bioactive compounds included caffeic acid, quercetin, chrysin, galangin, and pinobanksin (12). Therefore, the results of this studies indicated flavonoid compounds commonly present in the poplar type of China, Serbia, Italy, Slovenia and Germany propolis (23-27). 

In the current study, the antibacterial activity of EEIP of Ardabil and Polur propolis against *S. aureus *was higher than Chenaran, MoradBeyg, Kalaleh and Alborz proplis, that previously reported for Iranian propolis, while the growth inhibition zone against *E. coli* was less. Also, DEIP extracts indicated the growth inhibition zone less than this study against *S. aureus and E. coli *(16). Also in the previous study, EEIP of Ardabil inhibited the growth of oral streptococci with MIC values ranging from 3.12 to 100μg/ml and indicated good anti-biofilm activities against *streptococcus mutans *(28). The analysis of the effect of propolis on several bacterial species revealed that propolis is more active against gram-positive bacteria than gram-negative bacteria. It inhibited bacterial motility and enzymatic activity, which can lead to bacteriostatic activity. Also, at high concentrations, exhibit bactericidal capability against various bacterial species (29, 30). 

The results of the present study are consistent with the findings reported for the ethanol extract of propolis from Bulgaria, Greece, Turkey, Australian and Algeria, as samples showed a good antibacterial effect against *S. aureus*, but had a poor effect on gram-negative bacteria (31- 34). 

The studies indicate that flavonoids and derivatives can protect cells against cancer (35, 36). However, mechanisms of their protective effect on cells is unclear so far (37). Also, the inhibitory effect of propolis on the growth of cancer cell lines may be related to the production of reactive oxygen species (ROS) resulting to activation of apoptotic caspases in cancer cells (29). 

Accordingly, in previous studies we evaluated intracellular ROS induction mechanism involved in the anticancer effects of ethanolic extract of Iranian propolis against MCF-7 and RAW 264.7 cell lines using a flow cytometry method. In both studies, the levels of ROS increased significantly in cell lines in a dose-dependent manner compared with the control group (12, 38). Although in another study, we showed that Iranian propolis could inhibit the growth of KB and A431 cancer cells in a dose-dependent manner, but they had no effect on fibroblast cells compared to the control (28). In line with the results of other studies, cytotoxic activity of different extracts of propolis confirm our findings (39-46). 

Taken together, the chemical compositions of EEIP, DEIP and WEIP from two Iranian propolis samples showed the presence of compounds belonging to different groups such as aromatic acids, flavonoids and terpenes. EEIP of Ardabil sample had the highest percentage of flavonoids. Accordingly, it is expected that a high percentage of flavonoids in these extracts cause more biological activity against *S. aureus* and HN5 and LNCaP cell lines. In conclusion, we suggest that the synergistic effect of the main components of propolis is related to the increase of biological activity of propolis such as antimicrobial and cytotoxity. Further research is necessary to clarify the affecting mechanisms of the beneficial properties of propolis.

## References

[1] Wagh VD (2013). Propolis: a wonder bees product and its pharmacological potentials. Adv Pharmacol Sci.

[2] Kupchan SM (1970). Recent advances in the chemistry of tumor inhibitors of plant origin. Trans N Y Acad Sci.

[3] Zaccaria V, Curti V, Di Lorenzo A (2017). Effect of green and brown propolis extracts on the expression levels of micrornas, mrnas and proteins, related to oxidative stress and inflammation. Nutrients.

[4] Martinotti S, Ranzato E (2015). Propolis: a new frontier for wound healing?. Burns Trauma.

[5] Simone-Finstrom M, Borba RS, Wilson M, Spivak M (2017). Propolis counteracts some threats to honey bee health. Insects.

[6] Pietta PG, Gardana C, Pietta AM (2002). Analytical methods for quality control of propolis. Fitoterapia.

[7] Huang S, Zhang CP, Wang K, Li GQ, Hu FL (2014). Recent advances in the chemical composition of propolis. Molecules.

[8] Kosalec I, Bakmaz M, Pepeljnjak S, Vladimir-Knezevic S (2004). Quantitative analysis of the flavonoids in raw propolis from northern Croatia. Acta Pharm.

[9] Bankova VS, Castro SLd, Marcucci MC (2000). Propolis: recent advances in chemistry and plant origin. Apidologie.

[10] Ahangari Z, Naseri M, Vatandoost F (2018). Propolis: Chemical Composition and Its Applications in Endodontics. Iran Endod J.

[11] Kubiliene L, Laugaliene V, Pavilonis A (2015). Alternative preparation of propolis extracts: comparison of their composition and biological activities. BMC Complement Altern Med.

[12] Asgharpour F, Moghadamnia AA, Kazemi S (2018). Chemical Composition analysis and in vitro investigation of cytotoxic and antioxidative activities of iranian propolis against breast cancer cell line, MCF-7. Chem Select.

[13] Pasupuleti VR, Sammugam L, Ramesh N, Gan SH (2017). Honey, propolis, and royal jelly: a comprehensive review of their biological actions and health benefits. Oxid Med Cell Longev.

[14] Sena-Lopes Â, Bezerra FSB, das Neves RN (2018). Chemical composition, immunostimulatory, cytotoxic and antiparasitic activities of the essential oil from Brazilian red propolis. PLoS One.

[15] Sforcin JM, Bankova V (2011). Propolis: is there a potential for the development of new drugs?. J Ethnopharmacol.

[16] Afrouzan H, Tahghighi A, Zakeri S, Es-haghi A (2018). Chemical composition and antimicrobial activities of Iranian propolis. Iran Biomed J.

[17] Bauer AW, Kirby WMM, Sherris JC, Turck M (1966). Antibiotic susceptibility testing by a standardized single disk method. Am J Clin Pathol.

[18] CLSI Performance standards for antimicrobial susceptibility testing. 27th ed. CLSI Supplement M100S. Clinical and Laboratory Standards Institute, Wayne, IN. 2017.

[19] Alizadeh AM, Afrouzan H, Dinparast-Djadid N, Sawaya AC, Azizian S, Hemmati HR (2015). Chemoprotection of MNNG-initiated gastric cancer in rats using Iranian propolis. Arch Iran Med.

[20] Payandan E, Sayyed-alangi SZ, Shamloofar M, Koohsari H (2017). study of chemical composition and efficacy of different extracts of iranian propolis on the microbiological and sensory parameters of minced cyprinus carpio meat at 4°c storage. J Aquatic Food Prod Technol.

[21] Bankova V (2005). Recent trends and important developments in propolis research. Evid Based Complement Alternat Med.

[22] Ristivojevic P, Trifkovic J, Andric F, Milojkovic-Opsenica D (2015). Poplar-type propolis: chemical composition, botanical origin and biological activity. Nat Prod Commun.

[23] Pellati F, Orlandini G, Pinetti D, Benvenuti S (2011). HPLC-DAD and HPLC-ESI-MS/MS methods for metabolite profiling of propolis extracts. J Pharm Biomed Anal.

[24] Shi H, Yang H, Zhang X, Yu LL (2012). Identification and quantification of phytochemical composition and anti-inflammatory and radical scavenging properties of methanolic extracts of Chinese propolis. J Agric Food Chem.

[25] Ristivojevic P, Trifkovic J, Gasic U (2015). Ultrahigh-performance liquid chromatography and mass spectrometry (UHPLC-LTQ/Orbitrap/MS/MS) study of phenolic profile of Serbian poplar type propolis. Phytochemical analysis: PCA.

[26] Mavri A, Abramovic H, Polak T (2012). Chemical properties and antioxidant and antimicrobial activities of Slovenian propolis. Chem Biodivers.

[27] Chernetsova ES, Bromirski M, Scheibner O, Morlock GE (2012). DART-Orbitrap MS: a novel mass spectrometric approach for the identification of phenolic compounds in propolis. Anal Bioanal Chem.

[28] Asgharpour F, Moghadamnia AA, Zabihi E (2019). Iranian propolis efficiently inhibits growth of oral streptococci and cancer cell lines. BMC Complement Altern Med.

[29] Silva-Carvalho R, Baltazar F, Almeida-Aguiar C (2015). Propolis: a complex natural product with a plethora of biological activities that can be explored for drug development. Evid Based Complement Alternat Med.

[30] Mirzoeva OK, Grishanin RN, Calder PC (1997). Antimicrobial action of propolis and some of its components: the effects on growth, membrane potential and motility of bacteria. Microbiol Res.

[31] Seidel V, Peyfoon E, Watson DG, Fearnley J (2008). Comparative study of the antibacterial activity of propolis from different geographical and climatic zones. Phytotherapy Res.

[32] Dias LG, Pereira AP, Estevinho LM (2012). Comparative study of different Portuguese samples of propolis: Pollinic, sensorial, physicochemical, microbiological characterization and antibacterial activity. Food Chem Toxicol.

[33] Velikova M, Bankova V, Sorkun K, Houcine S, Tsvetkova I, Kujumgiev A (2000). Propolis from the Mediterranean region: chemical composition and antimicrobial activity. Z Naturforsch C J Biosci.

[34] Massaro CF, Katouli M, Grkovic T (2014). Anti-staphylococcal activity of C-methyl flavanones from propolis of Australian stingless bees (Tetragonula carbonaria) and fruit resins of Corymbia torelliana (Myrtaceae). Fitoterapia.

[35] Banskota AH, Tezuka Y, Kadota S (2001). Recent progress in pharmacological research of propolis. Phytother Res.

[36] Panche AN, Diwan AD, Chandra SR (2016). Flavonoids: an overview. J Nutr Sci.

[37] Hehlgans S, Lange I, Eke I, Kammerer B, Cordes N (2011). Human head and neck squamous cell carcinoma cell lines are differentially radiosensitised by the honeybee product Propolis. Int J Radiat Biol.

[38] Asgharpour F, Moghadamnia AA, Motallebnejad M, Nouri HR (2019). Propolis attenuates lipopolysaccharide-induced inflammatory responses through intracellular ROS and NO levels along with downregulation of IL-1β and IL-6 expressions in murine RAW 2647 macrophages. J Food Biochem.

[39] Devequi-Nunes D, Machado BAS, Barreto GA (2018). Chemical characterization and biological activity of six different extracts of propolis through conventional methods and supercritical extraction. PLoS One.

[40] Reis JHO, Barreto GA, Cerqueira JC (2019). Evaluation of the antioxidant profile and cytotoxic activity of red propolis extracts from different regions of northeastern Brazil obtained by conventional and ultrasound-assisted extraction. PLoS One.

[41] Utispan K, Chitkul B, Koontongkaew S (2017). Cytotoxic activity of propolis extracts from the stingless bee trigona sirindhornae against primary and metastatic head and neck cancer cell lines. Asian Pac J Cancer Prev.

[42] Barlak Y, Deger O, Colak M (2011). Effect of Turkish propolis extracts on proteome of prostate cancer cell line. Proteome Sci.

[43] Popolo A, Piccinelli LA, Morello S (2009). Antiproliferative activity of brown Cuban propolis extract on human breast cancer cells. Nat Prod Commun.

[44] Pratsinis H, Kletsas D, Melliou E, Chinou I (2010). Antiproliferative activity of Greek propolis. J Med Food.

[45] Eom HS, Lee EJ, Yoon BS, Yoo BS (2010). Propolis inhibits the proliferation of human leukaemia HL-60 cells by inducing apoptosis through the mitochondrial pathway. Nat Prod Res.

[46] Salehi M, Motallebnejad M, Moghadamnia AA (2017). An intervention airing the effect of iranian propolis on epithelial dysplasia of the tongue: a preliminary study. J Clin Diagn Res.

